# Evaluation of Health Science Students’ Health Fatalism and Perception Towards Patients With Epilepsy: A Cross-Sectional Global Study

**DOI:** 10.7759/cureus.30030

**Published:** 2022-10-07

**Authors:** Anas S Alyazidi, Osama Y Muthaffar, Fahad A Alotibi, Albatool Almubarak, Luca Tamai, Siba Z Takieddin, Maha Alghamdi, Yara K Alraddadi

**Affiliations:** 1 Department of Medicine, King Abdulaziz University Hospital, Jeddah, SAU; 2 Department of Medicine, King Abdulaziz University, Jeddah, SAU; 3 Department of Pediatrics, King Abdulaziz University Faculty of Medicine, Jeddah, SAU; 4 Department of Pediatrics, King Abdulaziz University Hospital, Jeddah, SAU; 5 Department of Psychology, University of Edinburgh, Edinburgh, GBR; 6 Department of Medicine and Surgery, Università Cattolica del Sacro Cuore, Rome, ITA; 7 Department of Internal Medicine, King Abdulaziz University Hospital, Jeddah, SAU

**Keywords:** italy, scotland, spain, saudi arabia, health fatalism

## Abstract

Introduction

The social acceptance of patients with epilepsy is largely determined by society's opinion of epilepsy; therefore, individuals with epilepsy could face prejudice and stigma as a result of negative impressions. Religious beliefs and mystical notions have been shown to influence attitudes toward epilepsy. Health fatalism could also be detrimental to society's and caregivers' approach toward such patients. In extreme settings, this could hinder them from obtaining an adequate treatment process.

Methods

A cross-sectional exploratory study was conducted from February 2022 to May 2022 in Saudi Arabia, Spain, Scotland, and Italy using an online questionnaire consisting of 33 questions concerning the Health Fatalism Scale (HFS), the Epilepsy Knowledge Scale (EKS), and the Epilepsy Attitude Scale (EAS).

Results

A total of 735 health science students (HSS) participated in the present study. The majority of participants were females (64.1%) while male participants represented 34.6% of the study. Health science students currently studying in Saudi Arabia represented the majority of participants with a percentage of 58.5%. Among the four countries, students in Saudi Arabia presented with the highest knowledge mean score. Students in Spain had the highest mean attitude score. Muslim students had the highest mean fatalism scores followed by Christian students.

Conclusion

In general, a high level of knowledge was observed among the participants, most notably, among Saudis who presented with the highest level of knowledge across the four countries. Regarding attitude, Spanish students presented the best attitude towards patients with epilepsy. Low fatalism scores were commonly observed across all countries regardless of their different demographic characteristics. Fatalism perception should be further detailed to ensure optimal services are delivered without prejudgment by future healthcare workers.

## Introduction

Epilepsy is a chronic brain condition that causes brief abnormal cerebral activity which can cause seizures, sensations, or loss of consciousness and affects over 70 million people worldwide [1,2]. Its global prevalence is reported to range between 4-10 per 1000 population [2]. The prevalence of epilepsy in Saudi Arabia, Italy, Spain, and Scotland has been reported to be 6.5 per 1000 population [3], 7.9 per 1000 population [4], 14.8 per 1000 population [5], and 10 per 1000 population [6], respectively. Epilepsy is linked to a variety of factors, including infectious, vascular, immune, structural, genetic, and metabolic etiologies [7]. Unfortunately, patients with epilepsy (PWE) could frequently face insufficient information and a lack of professional care [8] including poor understanding and communication as well as other barriers by caregivers [9]. The patients' families', and healthcare providers' knowledge of the disease's characteristics is critical to their success in adjusting to this lifelong condition [10]. The social acceptance of PWE is largely determined by society's opinion of epilepsy; most patients have significant difficulties in social acceptance. Sensitivity, fear of encountering a person with epileptic seizure, and helplessness are all recognized as negative elements influencing the attitudes towards epilepsy [11]. PWE face prejudice and stigma as a result of such attitudes, which have an impact on their education and professional lives [12]. Additionally, religious beliefs and mystical notions have been shown to influence attitudes toward epilepsy [13,14]. Previous literature reported that people who are religiously committed tend to demonstrate a more negative attitude towards the disorder [13]. Furthermore, people who blame epilepsy on spiritual causes were shown to have more negative attitudes [14]. Individuals employ a variety of spiritual ways to treat epilepsy besides medical treatment, including casting spells, praying, manufacturing amulets, and visiting holy places [15-18]. Moreover, fatalism is the concept which states that events in one's life are God's work and unavoidable such that they cannot be changed by any earthly power or effort; that they happen as a result of religious orientation [19,20]. Fatalism is popular in Muslim societies because fatalism is the primary religious belief; being one of Islam’s six pillars [21]. Fatalism says that God knows, sees, and manages everything, and that nothing can happen outside God's will [22]. Members of other religions including Christianity also believe that fatalism and religiosity directly and indirectly determine health outcomes, and disease management [23]. Individuals' attitudes and behaviors regarding health and disease are influenced by their religious beliefs. The view that health issues are largely beyond human control is known as health fatalism [24]. The goal of the religious health fatalism scale is to detect and possibly mitigate cognitive barriers to healthy behaviors, health services, and healthy lifestyle practices [25]. It has been noted that health fatalism has a detrimental impact on people's health behaviors and hinders them from actively participating in the diagnosis and treatment process [26-28]. Few studies have found that PWE are more fatalistic [29,30], believing that epilepsy is linked to religion, that it is viewed as a curse from God, and that its origins are spiritual in nature [13,16,31]. Patients' perspectives of their health and treatment may be influenced positively or negatively by the fatalist beliefs of health science students (HSS) who will provide care for PWE [32]. Students in the healthcare profession (i.e., HSS) should have the information and abilities required to fulfill the healthcare needs of PWE and their families, and education can thus aid in reducing the psychological difficulties that PWE might face [33]. HSS in institutional, clinical, community-based public health, or home-based care settings assume practitioner, leader, and researcher roles and responsibilities. In many settings, HSS deliver the largest volume of healthcare services to patients [8,34,35]. To ensure optimal healthcare delivery and support, HSS need to acquire at minimum a firm understanding and reliable knowledge of epilepsy as well as have positive attitudes towards PWE. As a result, in order to deliver more effective, high-quality healthcare to PWE, it's critical to assess HSS health fatalism, knowledge, and attitudes towards epilepsy, as well as the factors that influence these traits. The aim of this study is to evaluate HSS from different countries on their health fatalism with their epilepsy knowledge and attitudes score alongside analyzing their diverse sociodemographic characteristics.

## Materials and methods

Study design and setting

A cross-sectional exploratory study was conducted from February 2022 to May 2022 in Saudi Arabia, Spain, Scotland, and Italy. The study was in accordance with the Strengthening the Reporting of Observational Studies in Epidemiology (STROBE) guideline for cross-sectional studies.

Study population

HSS from various universities worldwide were the study's target population. The study was conducted using an electronic, self-administered questionnaire using Google Form in four locations that included the Faculty of Medicine at King Abdulaziz University, in Jeddah, Saudi Arabia; University of Deusto in Bilbao, Spain; University of Edinburgh in Edinburgh, Scotland; and Facoltà di Medicina e Chirurgia - Università Cattolica di Roma in Rome, Italy. The questionnaire was distributed online by the study’s co-authors who are affiliated with the aforementioned institutions.

Inclusion and exclusion criteria

The participants were HSS from different institutions and academic years. The study excluded incomplete results, duplicated responses, and students of specialties other than health sciences.

Sample size determination

Raosoft Sample Size Calculator software was employed to calculate the recommended sample size [36]. The required sample of HSS was found to be 385 participants after estimating the population size and response distribution. Also, they were chosen using a non-probability sampling technique to achieve a 95% confidence interval with a 5% margin of error.

Survey design and validation

The online questionnaire was in English and Arabic versions and consisted of 33 questions concerning the Health Fatalism Scale (HFS), the Epilepsy Knowledge Scale (EKS), and the Epilepsy Attitude Scale (EAS). Informed consent was obtained at the beginning of the survey, and participants were then asked several questions that were further divided into five sections. Section 1 identified the socio-demographic information such as gender, year of birth, marital status, religious affiliation, specialty, country of study, current year of study, and current grade-point average (GPA). Next, section 2 aimed to assess the familiarity of HSS with epilepsy using a questionnaire developed by Kılınçer et al. [37]. Questions included asking participants if they were “diagnosed with epilepsy”, “having a physician parent?”, “seeing a person with epileptic seizure”, “if they ever heard or read about epilepsy?”, and if they “know anyone with epilepsy?”.

Section 3 was designed to assess participants' current knowledge of epilepsy. The EKS was developed by Aydemir. The items on the knowledge scale yielded a Kuder-Richardson-20 internal consistency coefficient of 0.72 [38]. The knowledge scale consisted of 15 items, and the responses to the knowledge scale items were “true”, “false”, and “don’t know”. The items were designed to assess epilepsy medical knowledge, social aspects, and outside interventions during a seizure. Higher scores indicate a better understanding and greater knowledge of epilepsy.

Section 4 was concerned with evaluating participants’ current attitude towards epilepsy. The EAS was developed by Aydemir, and the Cronbach’s alpha was found to be 0.84 for the attitude scale [38]. The EAS consisted of 13 items which assessed respondents' cognitive beliefs about PWE, their affective reactions towards epilepsy and PWE, and their feelings about social contact with PWE. The responses to the attitude scale items were combined to create a 5-point Likert scale: “completely agree”, “agree”, “have no idea”, “disagree” and “completely disagree” with higher scores indicating more positive attitudes towards epilepsy and PWE.

Finally, section 5 aimed to assess participants’ current religious beliefs about epilepsy. The HFS was developed by Franklin et al. [25]. The scale used a Likert-style response scale ranging from 1 (strongly disagree) to 5 (strongly agree). As a result, higher scores on each scale indicate that the participant supported the items on each factor more strongly.

Ethical approval and study procedure

The Unit of Biomedical Ethics Research Committee at the Faculty of Medicine, King Abdulaziz University approved the study aim, protocol, survey, and procedure under reference number 169-22. The study adhered to the Declaration of Helsinki. All participants were informed of the aim of the study and confidentiality, and their rights to refuse participation or withdraw at any point during their participation. Consent was collected prior to enrollment. All information was kept private and anonymous. The questionnaire was distributed to students online as a direct web link to a Google Form on social media platforms.

Data analysis

Data were collected in a Microsoft Excel (Microsoft® Corp., Redmond, WA, USA) version 20 sheet. Descriptive statistical analysis was conducted using the Statistical Package for the Social Science (SPSS) version 21 (IBM Corp., Armonk, NY, USA). Measures of central tendency were calculated to describe quantitative variables, while frequencies and percentages were used for categorical variables. One-way analysis of variance (ANOVA) was used to analyze statistical differences between variables. All p-values <0.05, 95% confidence intervals were considered to be statistically significant.

## Results

Demographic characteristics

Table 1 represents the distribution of demographic data for this study. A total of 735 HSS participated in the present study. The majority of participants were females (64.1%) while male participants represented 34.6% and 1.4% of participants identified themselves as "other". The study included participants from their first academic year up to postgraduate students. First-year students constituted 4.8% of the total participants, second-year students represented 19.5%, third-year students were 17.8%, fourth-year students were 13.3%, fifth-year students were 10.9%, sixth-year students were the biggest group representing 21.4%, internship students were 2.7%, and postgraduates were 9.7%. HSS currently studying in Saudi Arabia represented the majority of participants - 430 (58.5%) students participated in the present study; 108 students were from Italy, 101 students were from Spain, and 96 students were from Scotland. The majority of students (81.1%) were single while the remaining either expressed to be married or in different forms of relationship. The religious affiliation of approximately half of the participants was Islam (58.8%), Atheists were 20.7%, Christians were 17.4%, and 3% of participants reported having different religious backgrounds and/or none. Students of medicine, psychology and medical rehabilitation represented 81.1% of participants while the remaining participants were students from different specialties including nursing, pharmacy, applied medical sciences, dentistry and medical laboratory. In response to students’ current grade average, 231 Saudi participants reported A±, 158 were B±, 33 were C± and eight were D±. Due to different grading systems in each country, we reported students' grades according to the adopted system which is represented in Table 1. To obtain participants’ socioeconomic status, the monthly household income was assessed. In Saudi Arabia, 19 participants had a monthly income of 3000 SAR or less, 20 had 3001-5000 SAR, 26 had 5001-7500 SAR, 47 had 7501-10,000 SAR, 87 had 10,001-15,000 SAR, 124 had 15,001-20,000 SAR and 107 had >20,001 SAR. Other household income data from Italy, Scotland and Spain are represented in Table 1.

**Table 1:** Demographic characteristics of the participants.

Item	n (%)
Gender	
Female	471 (64.1)
Male	254 (34.6)
Other	10 (1.4)
Current year of study	
First	35 (4.8)
Second	143 (19.5)
Third	131 (17.8)
Fourth	98 (13.3)
Fifth	80 (10.9)
Sixth	157 (21.4)
Internship	20 (2.7)
Postgraduate	71 (9.7)
Country of studying	
Saudi Arabia	430 (58.5)
Italy	108 (14.7)
Spain	101 (13.7)
Scotland	96 (13.1)
Relationship status	
Single	596 (81.1)
Married	44 (6.0)
Other	95 (12.9)
Religious affiliation	
Muslim	432 (58.8)
Atheist	152 (20.7)
Christian	128 (17.4)
Jewish	1 (0.1)
Buddhist	2 (0.3)
Spiritual	3 (0.4)
Pagan	3 (0.4)
Agnostic	9 (1.2)
None	5 (0.6)
Specialty	
Medicine	426 (58)
Psychology	115 (15.6)
Medical Rehabilitation	55 (7.5)
Nursing	34 (4.6)
Pharmacy	23 (3.1)
Applied Medical Sciences	16 (2.2)
Dentistry	15 (2.0)
Sciences	13 (1.8)
Medical laboratory	11 (1.5)
Public Health Sciences	8 (1.1)
Chemistry	9 (1.2)
Biology	6 (0.8)
Medical biotechnology	2 (0.3)
Midwifery	1 (0.1)
Social science	1 (0.1)
Current grade average	
Saudi Arabia	
A or A+ or A-	231 (31.4)
B or B+ or B-	158 (21.5)
C or C+ or C-	33 (4.5)
D or D+ or D-	8 (1.1)
Italy	
28-31	21 (2.9)
24-27	31 (4.2)
21-23	34 (4.6)
18-20	22 (3.0)
Spain	
A (Sobresaliente)	12 (1.6)
B (Notable)	77 (10.5)
C (Aprobado)	12 (1.6)
Scotland	
1st (90-100)	6 (0.8)
1st (80-89)	12 (1.6)
1st (70-79)	57 (7.8)
2.1 (60-69)	21 (2.9)
Monthly household income	
Saudi Arabia	
≤3000 Saudi Riyals	19 (2.6)
5000-3001 Saudi Riyals	20 (2.7)
7,500-5001 Saudi Riyals	26 (3.5)
10,000-7,501 Saudi Riyals	47 (6.4)
15,000-10,001 Saudi Riyals	87 (11.8)
20,000-15,001 Saudi Riyals	124 (16.9)
>20,001 Saudi Riyals	107 (14.6)
Scotland	
1000-2000 GBP	22 (3.0)
2000-3000 GBP	23 (3.1)
4000-5000 GBP	5 (0.7)
5000-7500 GBP	16 (2.2)
7500-10,000 GBP	7 (1.0)
>10,000 GBP	11 (1.5)
Italy and Spain	
1000 EUR	12 (1.6)
1000-2000 EUR	58 (7.9)
2000-3000 EUR	66 (9.0)
4000-5000 EUR	33 (4.5)
5000-7500 EUR	24 (3.3)
>10,000 EUR	16 (2.2)
1000 GBP	12 (1.6)

Familiarity with epilepsy

**Figure 1:**
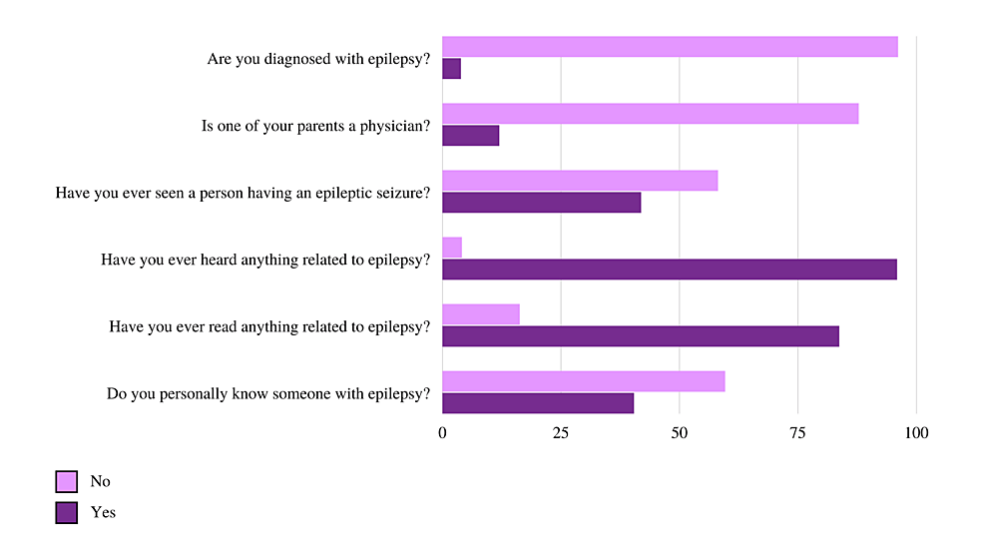
Participants’ familiarity with epilepsy.

A total of 706 participants (96.1%) were not diagnosed with epilepsy while the remaining 29 participants (3.9%) were diagnosed with some form of epilepsy. Only 88 participants had at least one of their parents as a physician compared to 645 (87.8%) who did not have a physician parent. When asked if they ever saw a person having an epileptic seizure, answers were split among participants where 427 (58.1%) answered “No” and 308 (41.9%) answered “Yes”. The majority of participants (95.9%) had heard something related to epilepsy while 4.1% did not. Furthermore, 615 respondents (83.7%) stated that they have read something related to epilepsy compared to 120 (16.3%) who did not. Finally, most participants (59.6%) stated that they have not personally known someone with epilepsy compared to 40.4% who stated otherwise. Detailed data concerning participants’ familiarity with epilepsy can be found in Figure 1.

Knowledge about epilepsy

A total of 619 participants (84.2%) think that most PWE can work. In addition, 582 (79.2%) believed that most children with epilepsy can go to public schools. Approximately half of the participants (55.1%) believed that PWE are not dangerous to others during a seizure, and only 29.8% of participants believed that they are dangerous. Furthermore, 631 participants (85.9%) think that there are some seizures that may last for a matter of seconds while 11.7% reported that they did not know if it is true or false. The majority of participants (78.4%) think it is true that for most PWE, seizures can be controlled with drugs. Additionally, 473 participants (64.4%) think that brain surgery can be used to treat epilepsy in certain cases. Nearly three-quarters (75.1%) of participants think that most PWE have average intelligence. A vast majority also think that PWE can be as successful at work as others. Furthermore, 546 participants (74.3%) believe that an epileptic seizure is caused by abnormal function of the nerve cells in the brain. A total of 335 participants (45.6%) answered “false” when asked if epilepsy is a kind of incurable disorder while 177 (24.1%) answered that this statement is “true”, and 223 participants answered with “I do not know”. The majority (74.3%) believed it is true that inadequate sleep, stress, and alcohol intake can cause seizures. Nearly half (56.2%) of the participants said it is false that seizures could be stopped by giving a person, with a seizure, an onion to smell during their seizure episode compared to 39.6% who said they do not know and 4.2% who said that this statement is true. Regarding seizure episodes, 78% of participants believe that some kinds of seizures can be hardly noticeable by others. Further detailed data on the knowledge section are represented in Table 2.

**Table 2:** Participants’ knowledge about epilepsy.

Item	n (%)
Most people with epilepsy can work	
False	4 (0.5)
True	619 (84.2)
I do not know	112 (15.2)
Most children with epilepsy can go to public schools	
False	36 (4.9)
True	582 (79.2)
I do not know	117 (15.9)
People with epilepsy can be dangerous to others during a seizure	
False	405 (55.1)
True	219 (29.8)
I do not know	111 (15.1)
Some seizures may last for a matter of seconds	
False	18 (2.4)
True	631 (85.9)
I do not know	86 (11.7)
For most people with epilepsy, seizures can be controlled with drugs	
False	29 (3.9)
True	576 (78.4)
I do not know	130 (17.7)
Brain surgery can be used to treat epilepsy in some cases	
False	24 (3.3)
True	473 (64.4)
I do not know	238 (32.4)
Most people with epilepsy have average intelligence	
False	19 (2.6)
True	552 (75.1)
I do not know	164 (22.3)
People with epilepsy can be as successful at work as others	
False	8 (1.1)
True	673 (91.6)
I do not know	54 (7.3)
An epileptic seizure is caused by an abnormal function of the nerve cells in the brain	
False	30 (4.1)
True	546 (74.3)
I do not know	159 (21.6)
Epilepsy is a kind of incurable disorder	
False	335 (45.6)
True	177 (24.1)
I do not know	223 (30.3)
Inadequate sleep, stress, and alcohol intake can cause a seizure	
False	27 (3.7)
True	546 (74.3)
I do not know	162 (22)
When you see a person having a seizure, you can stop the seizure by giving him/her an onion to smell	
False	413 (56.2)
True	31 (4.2)
I do not know	291 (39.6)
People with epilepsy can lead normal lives	
False	29 (3.9)
True	667 (90.7)
I do not know	39 (5.3)
Some kinds of seizures can be hardly noticed by others	
False	22 (3.0)
True	573 (78.0)
I do not know	140 (19.0)

Attitude towards epilepsy

A total of 384 participants (52.2%) chose “completely disagree” to a statement asking if they had seizures and whether they would hide it from their friends; 171 participants (23.3%) chose “disagree”, 88 participants (12%) said that they have no idea, 55 (7.5%) participants said that they agree to the statement, and 37 participants (5%) said they completely agree with the statement. When participants were asked whether they would stay away from their friends if they knew they have epilepsy, the majority of participants (85.9%) stated that they “completely disagree” with this statement. Only 2.1% stated either “agreed” or “completely agreed” with the statement. When participants were asked if they would object to working with someone who has epilepsy, the majority (76.1%) completely disagreed, 12.9% disagreed, 6.5% said they have no idea, and 4.4% completely agreed or only agreed. A total of 651 participants (88.6%) completely disagreed with a statement stating that they will be embarrassed if someone in their family had epilepsy. Furthermore, when asked if they would object to the marriage of their child with someone who has epilepsy, 49.9% completely disagreed with the statement, another 10.7% disagreed, 24.8% had no idea, 7.5% agreed, and 7.1% completely agreed. However, when asked if they, themselves were willing to marry someone who has epilepsy, 252 (34.3%) said they completely agree with the statement, 13.6% agreed, 28.8% said they have no idea, 11% disagreed, and 12.2% completely disagreed. When asked if they would not trust a doctor with epilepsy if they knew of his condition, around 66.4% said that they completely disagree with the statement, 16.6% disagreed and 12.9% had no idea. The majority (79.2%) completely disagreed with the statement that they prefer to stay away from someone with epilepsy with only 3% either completely agreed or agreed. Participants were also asked if having epilepsy is something to be embarrassed about, to which 618 participants (84.1%) reported that they completely disagree. Moreover, participants were asked if they would feel uncomfortable working with someone who has epilepsy, to which 555 participants (75.5%) completely disagreed, 13.7% disagreed, 4.9% had no idea, 2.9% agreed and 3% completely agreed. When asked if the participants think PWE are frightening, 78.5% and 12.1% completely disagreed and disagreed, respectively. Participants were further asked if they think PWE are not physically attractive and the majority (87.3%) completely disagreed. Only 0.8% completely agreed or agreed with this statement. Finally, participants were asked if they feel uncomfortable staying alone with someone who has epilepsy, 67.9% completely disagreed. Further detailed data on the attitude section are represented in Table 3.

**Table 3:** Participants’ attitude towards epilepsy.

Item	n (%)
If I had epilepsy I would hide it from my friends	
Completely disagree	384 (52.3)
Disagree	171 (23.3)
I have no idea	88 (11.9)
Agree	55 (7.5)
Completely agree	37 (5.0)
I would stay away from a friend if I knew she/he had epilepsy	
Completely disagree	631 (85.9)
Disagree	61 (8.3)
I have no idea	28 (3.7)
Agree	7 (1.0)
Completely agree	8 (1.1)
I would object to work with someone who has epilepsy	
Completely disagree	559 (76.2)
Disagree	95 (12.9)
I have no idea	48 (6.5)
Agree	15 (2.0)
Completely agree	18 (2.4)
I would be embarrassed if someone in my family had epilepsy	
Completely disagree	651 (88.6)
Disagree	55 (7.4)
I have no idea	19 (2.6)
Agree	3 (0.4)
Completely agree	7 (1.0)
I would object to the marriage of my child with someone who has epilepsy	
Completely disagree	367 (49.9)
Disagree	79 (10.7)
I have no idea	182 (24.8)
Agree	52 (7.1)
Completely agree	55 (7.5)
I would marry someone who has epilepsy	
Completely disagree	90 (12.2)
Disagree	81 (11.0)
I have no idea	212 (28.9)
Agree	100 (13.6)
Completely agree	252 (34.3)
I would not trust a doctor with epilepsy, if I knew of her/his illness	
Completely disagree	488 (66.4)
Disagree	122 (16.6)
I have no idea	95 (12.9)
Agree	13 (1.8)
Completely agree	17 (2.3)
I prefer to stay away from someone with epilepsy	
Completely disagree	582 (79.2)
Disagree	88 (12.0)
I have no idea	43 (5.8)
Agree	19 (2.6)
Completely agree	3 (0.4)
Having epilepsy is something to be embarrassed about	
Completely disagree	618 (84.1)
Disagree	49 (6.7)
I have no idea	36 (4.8)
Agree	10 (1.4)
Completely agree	22 (3.0)
I feel uncomfortable working with someone who has epilepsy	
Completely disagree	555 (75.5)
Disagree	101 (13.7)
I have no idea	36 (4.9)
Agree	21 (2.9)
Completely agree	22 (3.0)
I think patients with epilepsy are frightening	
Completely disagree	577 (78.5)
Disagree	89 (12.1)
I have no idea	53 (7.2)
Agree	9 (1.2)
Completely agree	7 (1.0)
I think people with epilepsy are not physically attractive	
Completely disagree	642 (87.3)
Disagree	48 (6.5)
I have no idea	39 (5.4)
Agree	3 (0.4)
Completely agree	3 (0.4)
I feel uncomfortable staying alone with someone who has epilepsy	
Completely disagree	499 (67.9)
Disagree	99 (13.5)
I have no idea	82 (11.2)
Agree	29 (3.9)
Completely agree	26 (3.5)

Scores assessment

Table 4 shows a detailed description of the participants' scores. Across the four countries, students in Saudi Arabia presented with the highest knowledge mean score (10.31). Spain and Scotland presented almost equal mean scores of 10.25 and 10.24, respectively. Italy had the lowest mean score for the knowledge section with a mean of 9.68. When assessing participants’ knowledge scores according to their year of study, sixth-year students presented with the highest mean score while second-year students had the lowest knowledge mean score. Interns and postgraduate students had a relative score. When investigating students’ current grade average, the mean knowledge score (MKS) correlated positively with students' grades. Saudi students who reported the lowest grade average (i.e., D or D+ or D-) had the lowest MKS (8.25) compared to students who reported higher grades and had a higher mean knowledge score. Similarly, this was the case for participants from Scotland as students who reported the lowest grades (i.e., 2.1 (60-69)) had the lowest MKS (9.38). Data from Spain presented similar findings to those of Saudi Arabia and Scotland. However, in Italy, the score correlates positively with the student's current average grade. Students in Italy who reported having a current grade average of 21-23 had the highest MKS compared to those who had a current grade average of 24-27 and 28-31 and had an MKS of 9.19 and 9.90, respectively. Moreover, when assessing the level of income compared to the knowledge score, results showed that, in general, the higher the participants' household income the higher their MKS.

**Table 4:** Participants’ demographic characteristics adjusted to the knowledge score using logistic regression model

					95% Confidence Interval for Mean	
	Demographic characteristics	n	Mean	S.D.	Lower Bound	Upper Bound
Knowledge score	Country of studying					
	Saudi Arabia	430	10.31	2.77	10.05	10.57
	Italy	108	9.68	2.022	9.29	10.06
	Spain	101	10.25	2.52	9.75	10.74
	Scotland	96	10.24	2.31	9.77	10.71
	Year of study					
	1	35	10.17	2.57	9.29	11.06
	2	143	8.91	2.84	8.45	9.39
	3	131	9.60	2.68	9.14	10.07
	4	98	10.12	2.85	9.55	10.69
	5	80	10.75	2.29	10.24	11.26
	6	157	11.71	1.17	11.53	11.90
	Internship	20	10.30	2.43	9.16	11.44
	Postgraduate	71	10.00	2.42	9.43	10.57
	Current grade average					
	A or A+ or A-	231	10.29	2.69	9.94	10.63
	B or B+ or B-	158	10.42	2.93	9.96	10.88
	C or C+ or C-	33	10.42	2.18	9.65	11.20
	D or D+ or D-	8	8.25	3.85	5.04	11.46
	1st (90-100)	6	10.83	1.60	9.15	12.51
	1st (80-89)	12	11.17	2.33	9.69	12.65
	1st (70-79)	57	10.30	2.18	9.72	10.88
	2.1 (60-69)	21	9.38	2.64	8.18	10.58
	28-31	21	9.90	2.55	8.75	11.06
	24-27	31	9.19	2.12	8.42	9.97
	21-23	34	10.00	1.65	9.42	10.58
	18-20	22	9.64	1.84	8.82	10.45
	A (Sobresaliente)	12	10.50	1.68	9.43	11.57
	B (Notable)	77	10.34	2.52	9.77	10.91
	C (Aprobado)	12	9.42	3.20	7.38	11.45
	Monthly household income					
	3000 Saudi Riyals	19	8.42	2.76	7.09	9.75
	5000-3001 Saudi Riyals	20	9.20	4.05	7.30	11.09
	7,500-5001 Saudi Riyals	26	9.69	2.75	8.58	10.80
	10,000-7,501 Saudi Riyals	47	9.89	3.26	8.94	10.85
	15,000-10,001 Saudi Riyals	87	10.63	2.44	10.11	11.15
	20,000-15,001 Saudi Riyals	124	10.80	2.44	10.37	11.23
	>20,001 Saudi Riyals	107	10.36	2.72	9.83	10.88

Assessing the attitude scores presented in Table 5 revealed that students in Spain had the highest mean attitude score (MAS) reaching 53.53 which represents the most positive attitude towards epilepsy. Scotland was second with an MAS of 51.72, Saudi Arabia ranked third with an MAS of 51.49, and finally, Italy had the lowest MAS with a score of 50.45. When assessing the fatalism score, religious affiliation was included in the analysis given its significant relevance to the fatalism section. Students in Saudi Arabia had the highest mean fatalism score (MFS) at 1.77, students in Italy ranked second with an MFS of 1.45, students in Scotland ranked third with an MFS of 1.28, and finally, students in Spain had the lowest MFS with a score of 1.08. Furthermore, Muslim students had the highest MFS reaching 1.76, followed by Christian students with an MFS of 1.54, and Atheists with 1.10. MFS for other religious affiliations were invalid due to the lack of respondents within this category. Comparing the MFS to participants’ grade average, Saudi students with the lowest grade average had the highest MFS which reached 2.38. Similarly, this was the case for students in Scotland where students with the lowest grade average had the highest MFS (1.38).

**Table 5:** Participants’ demographic characteristics adjusted to the attitude score using logistic regression model.

					95% Confidence Interval for Mean	
	Demographic characteristics	n	Mean	S.D.	Lower Bound	Upper Bound
Attitude score	Country of studying					
	Saudi Arabia	430	51.49	5.99	50.92	52.06
	Italy	108	50.45	4.41	49.61	51.29
	Spain	101	53.53	2.81	52.98	54.09
	Scotland	96	51.72	3.72	50.97	52.47
	Year of study					
	1	35	49.51	4.51	47.97	51.06
	2	143	50.06	5.85	49.09	51.02
	3	131	50.76	4.72	49.94	51.57
	4	98	50.83	5.92	49.64	52.01
	5	80	52.08	4.59	51.05	53.10
	6	157	54.57	2.52	54.18	54.97
	Internship	20	49.95	8.43	46.00	53.90
	Postgraduate	71	52.23	5.48	50.93	53.52
	Current grade average					
	A or A+ or A-	231	51.45	5.69	50.71	52.19
	B or B+ or B-	158	51.60	6.24	50.62	52.58
	C or C+ or C-	33	51.61	6.68	49.24	53.97
	D or D+ or D-	8	50.00	7.27	43.92	56.08
	1st (90-100)	6	46.50	2.66	43.70	49.30
	1st (80-89)	12	53.00	2.66	51.31	54.69
	1st (70-79)	57	52.33	3.70	51.35	53.32
	2.1 (60-69)	21	50.81	3.25	49.33	52.29
	28-31	21	49.95	4.91	47.71	52.19
	24-27	31	52.32	3.61	51.00	53.65
	21-23	34	49.29	4.20	47.83	50.76
	18-20	22	50.09	4.69	48.01	52.17
	A (Sobresaliente)	12	53.83	2.76	52.08	55.59
	B (Notable)	77	53.40	2.93	52.74	54.07
	C (Aprobado)	12	54.08	2.11	52.74	55.42
	Monthly household income					
	3000 Saudi Riyals	19	50.00	6.95	46.65	53.35
	5000-3001 Saudi Riyals	20	50.25	6.69	47.12	53.38
	7,500-5001 Saudi Riyals	26	51.50	3.25	50.19	52.81
	10,000-7,501 Saudi Riyals	47	49.68	7.41	47.50	51.86
	15,000-10,001 Saudi Riyals	87	51.83	6.66	50.41	53.25
	20,000-15,001 Saudi Riyals	124	52.42	4.73	51.58	53.26
	>20,001 Saudi Riyals	107	51.43	6.10	50.26	52.60

However, differences were not significant among students in Italy as they were in Saudi Arabia and Scotland. More details are presented in Table 6.

**Table 6:** Participants’ demographic characteristics adjusted to the fatalism score using logistic regression model.

					95% Confidence Interval for Mean	
	Demographic characteristics	n	Mean	S.D.	Lower Bound	Upper Bound
Fatalism score	Country of studying					
	Saudi Arabia	430	1.77	1.25	1.65	1.89
	Italy	108	1.45	.80	1.30	1.61
	Spain	101	1.08	.44	.99	1.17
	Scotland	96	1.28	.63	1.15	1.41
	Religious affiliation					
	Muslim	432	1.76	.84	1.64	1.87
	Christian	128	1.54	1.24	1.39	1.69
	Atheist	152	1.10	.43	1.03	1.17
	Jewish	1	Not valid			
	Buddhist	2				
	Spiritual	3				
	Pagan	3				
	Nothing	5				
	Agnostic	9				
	Current grade average					
	A or A+ or A-	231	1.79	1.22	1.64	1.95
	B or B+ or B-	158	1.63	1.19	1.45	1.82
	C or C+ or C-	33	2.12	1.62	1.55	2.69
	D or D+ or D-	8	2.38	1.51	1.12	3.63
	1st (90-100)	6	1.17	.41	.74	1.60
	1st (80-89)	12	1.25	.45	.96	1.54
	1st (70-79)	57	1.26	.67	1.09	1.44
	2.1 (60-69)	21	1.38	.67	1.08	1.69
	28-31	21	1.33	.48	1.11	1.55
	24-27	31	1.03	.18	.97	1.10
	21-23	34	1.60	.75	1.30	1.82
	18-20	22	2.00	1.23	1.45	2.55
	A (Sobresaliente)	12	1.00	.00	1.00	1.00
	B (Notable)	77	1.10	.50	.99	1.22
	C (Aprobado)	12	1.00	.00	1.00	1.00
	Monthly household income					
	3000 Saudi Riyals	19	2.47	1.47	1.77	3.18
	5000-3001 Saudi Riyals	20	2.10	1.48	1.41	2.79
	7,500-5001 Saudi Riyals	26	1.65	1.20	1.17	2.14
	10,000-7,501 Saudi Riyals	47	2.36	1.47	1.93	2.79
	15,000-10,001 Saudi Riyals	87	1.60	1.04	1.38	1.82
	20,000-15,001 Saudi Riyals	124	1.47	1.05	1.28	1.66
	>20,001 Saudi Riyals	107	1.84	1.32	1.59	2.09

**Figure 2:**
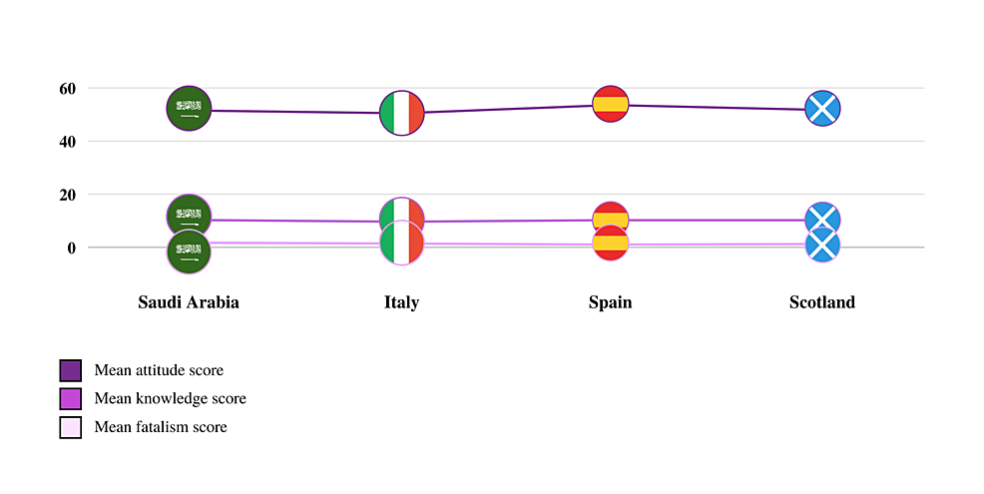
Mean knowledge, attitude and fatalism scores according to participants’ country of study.

In Spain, the scores did not significantly differ across students with different grade average. Finally, students in Saudi Arabia with the lowest household income presented with the highest MFS which was 2.47. Students in Saudi Arabia with a monthly household income of less than 3000 SAR had the single highest MFS group compared to groups of higher household income. Further details on the MFS in relation to the monthly household income are represented in Table 6. Mean knowledge, attitude and fatalism scores according to participants’ country of studies are also summarized in Figure 2.

Differences in scores across all participating countries

Differences in knowledge, attitude and fatalism among HSS in different countries were assessed using Tukey’s Honestly Significant Difference (Tukey’s HSD). The differences in the MKS were significant between HSS in Saudi Arabia and Italy (P=0.02) (Table 7).

**Table 7:** Differences in participants’ knowledge score according to the country of study using post hoc comparison methods. The mean difference is significant at the 0.05 level. HSD: Honestly Significant Difference; LSD: Least Significant Difference.

	(I) Country of study	(J) Country of study	Mean Difference (I-J)	SE	P	95% Confidence Interval	
						Lower Bound	Upper Bound
Tukey HSD	Saudi Arabia	Italy	.63	.28	.104	-.08	1.35
		Scotland	.07	.29	1.00	-.68	.82
		Spain	.06	.29	1.00	-.67	.80
	Italy	Saudi Arabia	-.63	.28	.10	-1.35	.08
		Scotland	-.56	.36	.40	-1.50	.37
		Spain	-.57	.36	.38	-1.49	.35
	Scotland	Saudi Arabia	-.07	.29	1.00	-.82	.68
		Italy	.56	.36	.40	-.37	1.50
		Spain	-.01	.37	1.00	-.96	.94
	Spain	Saudi Arabia	-.06	.29	1.00	-.80	.67
		Italy	.57	.36	.38	-.35	1.49
		Scotland	.01	.37	1.00	-.94	.96
LSD	Saudi Arabia	Italy	.63*	.28	.02	.09	1.18
		Scotland	.07	.29	.81	-.50	.64
		Spain	.06	.29	.83	-.50	.62
	Italy	Saudi Arabia	-.63*	.28	.02	-1.18	-.09
		Scotland	-.56	.36	.12	-1.28	.15
		Spain	-.57	.36	.11	-1.27	.13
	Scotland	Saudi Arabia	-.07	.29	.81	-.64	.50
		Italy	.56	.36	.12	-.15	1.28
		Spain	-.008	.37	.98	-.73	.71
	Spain	Saudi Arabia	-.06	.29	.83	-.62	.50
		Italy	.57	.36	.11	-.13	1.27
		Scotland	.01	.37	.98	-.71	.73

Furthermore, comparing MAS, analysis shows a significant statistical difference in the MAS when comparing HSS in Spain and Italy (p=0.000). Tukey’s HSD comparisons indicated that participants from Italy scored on average 3.08 points lower on the MAS compared to participants from Spain (95% CI [-4.92, -1.24]). Similarly, Tukey’s HSD comparisons indicated that participants from Saudi Arabia scored on average 2.04 points lower on the MAS compared to participants from Spain (95% CI [-3.52, -0.57]), differences in the MAS were also significant between HSS in Saudi Arabia and Spain (p=0.002) (Table 8). Moreover, means for groups in homogeneous subsets showed statistical significance in MAS between HSS in Spain and other countries.

**Table 8:** Differences in participants’ attitude score according to the country of study using post hoc comparison methods. The mean difference is significant at the 0.05 level. HSD: Honestly Significant Difference; LSD: Least Significant Difference

	(I) Country of study	(J) Country of study	Mean Difference (I-J)	SE	P	95% Confidence Interval	
						Lower Bound	Upper Bound
Tukey HSD	Saudi Arabia	Italy	1.04	.557	.245	-.40	2.47
		Scotland	-.23	.584	.980	-1.73	1.28
		Spain	-2.04*	.57	.002	-3.52	-.57
	Italy	Saudi Arabia	-1.04	.56	.245	-2.47	.40
		Scotland	-1.27	.73	.302	-3.13	.60
		Spain	-3.08*	.72	.000	-4.92	-1.24
	Scotland	Saudi Arabia	.23	.58	.980	-1.28	1.73
		Italy	1.27	.73	.302	-.60	3.13
		Spain	-1.82	.74	.067	-3.7	.08
	Spain	Saudi Arabia	2.04*	.57	.002	.57	3.52
		Italy	3.08*	.72	.000	1.24	4.92
		Scotland	1.82	.74	.067	-.08	3.71
LSD	Saudi Arabia	Italy	1.04	.56	.063	-.05	2.13
		Scotland	-.23	.58	.696	-1.37	.92
		Spain	-2.04*	.57	.000	-3.17	-.92
	Italy	Saudi Arabia	-1.04	.56	.063	-2.13	.06
		Scotland	-1.27	.73	.082	-2.69	.16
		Spain	-3.08*	.72	.000	-4.49	-1.68
	Scotland	Saudi Arabia	.23	.58	.696	-.91	1.37
		Italy	1.27	.73	.082	-.16	2.69
		Spain	-1.82*	.74	.014	-3.26	-.37
	Spain	Saudi Arabia	2.04*	.57	.000	.92	3.17
		Italy	3.08*	.72	.000	1.68	4.49
		Scotland	1.82*	.74	.014	.37	3.26

## Discussion

Epilepsy remains a condition characterized by a lack of understanding, false assumptions, prejudices and social stigma. Changes in knowledge, attitudes and beliefs are slow to come about in society as a whole. It has been demonstrated in this study that as education and information levels rise, fatalism declines. This is in line with other studies which discovered that as the number of study years increased, people shifted away from thinking that disasters are fate [39]. In this study, the perception, which included familiarity, knowledge, attitude, and fatalism, was for the first time explored across four populations of similar characteristics and diverse religious and educational backgrounds.

Epilepsy familiarity

Upon assessing the familiarity with epilepsy, a small percentage of HSS reported having epilepsy, and a small group expressed having a physician parent. Nonetheless, the majority of HSS were found to be very familiar with the condition in several aspects. However, a higher proportion of HSS have never seen a person with epileptic seizure. As a future caregiver, it is important to be familiarized with the agitation and reflexes experienced by the epileptic patient to be able to provide an adequate resuscitation. First aid courses and in-clinic exposure and even online visual materials can aid in reducing this gap. The percentage of HSS students who have never seen a person with epilepsy (58.1%) was very similar to the findings in the literature (57.3%) nine years ago [40]. The difference, however, was in the targeted population of which the latter results came from the general public. Higher exposure to epileptic seizures is, nevertheless, more important to HSS than the general public. Surprisingly, a very high majority of participants had heard something about epilepsy (95.9%). This significant difference was higher than studies conducted on the general public (71.53%) [41]. Other studies reported different findings that included higher percentages of familiarity by hearing or reading about epilepsy among the general public (94.79%) [41]. Furthermore, this study presented evidence that HSS would more likely hear about epilepsy rather than read about it. The drop in percentage between the two modalities requires attention as recent investigations suggested that medical students, specifically, must build strong reading and study habits early, as it will guide them towards more reliable resources and eventually enable them to provide a safer care to patients [42].

Epilepsy knowledge

The most significant aspects concerning HSS knowledge of epilepsy have been outlined. The information was gathered to assess the differences that exist between students from different countries, from different years of study and between students who report different grade average and different monthly household income. All the information discussed in the following subsections is detailed in Table 4. The study's analysis revealed high levels of knowledge about epilepsy in HSS. Our findings were consistent with other studies where students possessed excellent levels of knowledge in their last years of study in the health departments of universities in several nations (Brazil, the United States of America, Portugal, Argentina, and South Africa) [43].

Knowledge on epilepsy and country of study

The general knowledge of health students about epilepsy can be considered high. However, students from Italy notably seem to have a slightly poorer degree of knowledge than those studying in Saudi Arabia, Spain and Scotland. Apparently, Italian students lack knowledge on epilepsy and this could be attributed to the fact that in Italy it is common practice to only research extensively on those specific diseases one daily faces with in his or her career as a doctor. In other words, students will usually learn general information about different conditions but only research in a specific field such as the one represented by epilepsy if and when they start their career as, for instance, a neurologist. We must in any case admit such a difference left us without any other explanation. Another study [44] also found that Italian students are not adequately prepared for epilepsy, although the study did not compare the results with those from any other country. To the extent of our knowledge, no other research was ever conducted to confirm such a difference between the countries within the scope of this present study.

Knowledge on epilepsy and year of study

Remarkably, in contrast to what was seen by Turan et al. [32], we found that first-year medicine students seem to have higher knowledge than those in their second, third and fourth years. Nevertheless, students in their fifth or sixth year have a knowledge score which is higher than the one of the students from any other year. While we cannot say what is causing this difference, it is conceivable that those who are about to start their path of education as future medical doctors spend some free time reading about well-known diseases and informing themselves. The information that first-year students acquire by themselves for their own interest might then be lost as they start studying pre-clinical subjects which might be resulting in the decrease of knowledge that we observed. Further research is required to confirm this speculation. It goes without saying that older students, who should have studied subjects like neurology, have a better knowledge on epilepsy, as shown by Ezeala-Adikaibe et al. [45] where this particular factor was investigated. The point is consistent with what was reported by Souza et al. [43]. The difference between what we found and what was reported by Turan et al. [32], is possibly due to the fact that the target of the latter study was a group of nursing students, whose main interest might be in matters different from epilepsy, or more generally medical disorders. Another study on students indicated that those who have previously encountered epileptic cases had a high level of awareness of the condition [46]. The current study's results correspond with the body of literature as HSS had extremely positive opinions toward epilepsy which was also seen in the literature [47].

Knowledge on epilepsy and different personal attributes: grade average and monthly income

The link between the degree of knowledge and grade average is coherent and in line with the results reported by a similar study [48]: the higher the grade average, the higher the knowledge students show to have. Whilst what we have just stated tends to be generally true, it must also be noted that this trend is not particularly evident when looking at the results reported by Italian students. In other words, Italian students’ knowledge score does not significantly differ between those with a higher grade average (28-31 and 24-27) and those with the lower one (21-23 and 18-20). As for the link between knowledge score and monthly household income, the data from Saudi Arabia shows that the knowledge score tends to get better as students come from families with higher income. This piece of evidence is arguably attributable to the fact that those coming from families with higher household income might have some medical background and have therefore learnt reliable information on epilepsy independent of their academic studies.

Epilepsy attitude

The attitude of students towards epilepsy was shown to be positive in a study by Unsar et al. [47], and a study on medical students claimed high epilepsy attitude scale (EAS) scores. According to numerous research findings [46,49], students exhibit favorable opinions toward those who have epilepsy. The findings of this study supported previous research demonstrating that students studying health sciences had valid opinions towards epilepsy. In this study, female students, fifth- and sixth-year students, and those with epilepsy in their close social circles or immediate surroundings all had high EAS scores. Another study found that female students and those who had previously cared for epileptic patients had high mean EAS scores [50]. A third study found that students with epilepsy in their families, those who had seen epileptic seizures, and those who had previously cared for epileptic patients had more positive views [51]. A study done on nursing students and laboratory assistants found that those who had previously dealt with epileptic cases exhibited more positive attitudes [46]. The knowledge and attitudes of students appeared to significantly improve over time and through exposure (i.e., through frequent encounters with PWE) [49]. Students who provided care to PWE had more accurate information and more favorable attitudes than students who did not. The social roles played by the students and the frequency of their interactions with PWE may have had a favorable impact on their perceptions. As their knowledge levels rose, students' views toward epilepsy tended to improve similarly to the findings of Vodougnon et al. [52]. Numerous research have demonstrated that students' views towards epilepsy improved when their knowledge scores rose [13,47,53]. Our study's findings verified prior studies' findings, which showed that attitude ratings were positively impacted by having more knowledge regarding epilepsy.

Epilepsy fatalism

While for years researchers have primarily focused on assessing the knowledge, and attitudes towards patients with epilepsy, this study presented new aspects by including a multinational sample. Our study also assessed the health fatalism and influence of religious beliefs on future caregivers - only a few researchers have done so. According to the current study's findings, Saudi students had the highest MFS (1.77), and as expected, Muslim students had the highest MFS reaching 1.76. We compared our findings with studies conducted on different groups as the literature lack studies assessing HSS health fatalism towards patients with epilepsy. Our results are consistent with a previous study reported by Sülü Uğurlu et al. which indicated that religious beliefs encourage a fatalistic mindset, and the fact that Islamic belief includes the fate principle makes the fatalistic mindset likely among Muslims [21]. The observed increase in the MFS among Muslim students could be attributed to the idea that Islamic beliefs, or religiosity in general can eventually lead to fatalism. The faith that God ultimately controls fate was simultaneously confirmed by our Muslim sample. Arguably, for many of them, this has roots in the Islamic doctrine of predestination according to which an omniscient and controlling deity is compatible. Moreover, the concept of fate is one of Islam's six pillars. Fatalism, which also plays a significant role in the Islamic faith, affirms that nothing can occur against God's will [22]. The extent of fatalism’s impact on HSS remains an active area of research with psychological, cultural and social determinants that are not fully understood yet. Theological and teleological considerations centered around God seem to be very crucial for Muslims compared to other religious groups [54]. This finding aids in interpreting the current study's findings and the fatalism assessment, especially among Muslim participants. The variation in views among different religious groups, especially easterners compared to westerners, can be attributed to the way of thinking under uncertainty which can be supported by the findings of Guo et al. [55].

Limitations

Due to the study's novelty, the lack of previous studies nationwide made it difficult to compare the findings to others. The study design (cross-sectional) also presents potential selection bias and unequal samples across different groups of population. These limitations, however, were addressed by increasing the sample size. Furthermore, the fatalism section's components were adopted from a validated scale and formulated on the basis of our study's objectives.

## Conclusions

In conclusion, this study assessed the fatalism, knowledge and attitude of HSS across four countries. While very few studies assessed the same aspects, this study serves as the first international, interreligious assessment to have ever been conducted. In general, participants presented with a high level of knowledge. The exact variation in the knowledge levels was explored using multiple tools yielding novel descriptive data. HSS in Saudi Arabia presented the best level of knowledge across the four countries. Regarding attitude, Spanish students presented the best attitude towards PWE. The positive attitude presented by Spanish participants requires understanding to reduce stigmatization towards patients with epilepsy. A possible approach for future research is studying whether participants’ attitudes would differ depending on how epilepsy is presented; as a condition (epilepsy) or as a patient with epilepsy. Low fatalism scores were commonly observed across all countries regardless of their different demographic characteristics. The biggest gap in fatalism scores within a single population was observed among Saudis more significantly than other populations. To understand this variation, it is recommended to conduct social and community-based studies that address all social and cultural aspects. Understanding cultural influences on these populations could aid in more in-depth interpretation of this study's findings. Moreover, fatalism perception should be further detailed to ensure optimal services are delivered without prejudgment by future healthcare workers. Educational programs should be implemented to raise knowledge and awareness towards epilepsy and limit inequalities.
